# An Evaluation of Intra-articular Pathologies Accompanying Tibial Plateau Fractures Postoperatively

**DOI:** 10.7759/cureus.71350

**Published:** 2024-10-13

**Authors:** Sefa Erdem Karapinar, Salih Korkmaz, Recep Dincer

**Affiliations:** 1 Department of Orthopedics and Traumatology, Suleyman Demirel University, Isparta, TUR; 2 Department of Orthopedics and Traumatology, Davraz Yasam Hospital, Isparta, TUR

**Keywords:** meniscus and ligament pathologies, mri, osteoarthritis, tibia plateau fracture, worms

## Abstract

Introduction and aim: Tibial plateau fractures are one of the most complex and common intra-articular fractures encountered in trauma surgery. This study aims to evaluate the MRI intra-articular findings of patients with tibial plateau fractures treated surgically and the functional and radiological changes after surgical treatment.

Materials and methods: A total of 91 patients admitted to our clinic between 2010 and 2019 and operated for tibial plateau fracture were identified from the hospital information system. Patients were excluded from the study if aged <18 years, if the radiographs and MRIs were not suitable for the measurements, or if they could not be contacted by telephone. The study was completed with 49 operated knees and 49 healthy knees of 49 patients. The patients were evaluated with respect to demographic characteristics, Knee Society Knee Score (KSKS), Knee Society Function Score (KSFS), Knee Injury and Osteoarthritis Outcome Score (KOOS) functional and clinical scores, Rasmussen, Kellgren-Lawrence, and Whole-Organ Magnetic Resonance Imaging Score (WORMS) radiological scoring, and meniscus and ligament pathologies examined on the follow-up MR images.

Results: The patients comprised 39 males and 10 females with a mean age of 45.9±11.89 years, and a mean follow-up of 58.2±30.7 months. The fractures were seen to be 49% (n=24)in the lateral plateau, 6.1% (n=4) in the medial plateau, and 42.9% (n=21) were bicondylar fractures involving both plateaux. The KSKS knee score was satisfactory in 85.7% of the patients, the KSFS functional score in 89.7%, and the Rassmussen radiological score in 93.8%. In the KOOS subjective questionnaire, the mean points obtained were 430.7, and the mean total WORMS points were 34.2. According to the Kellgren-Lawrence grades, severe osteoarthritis (grade 3-4) developed in 18.3% (n=9) of the patients.

Conclusion: Meniscus injuries that are overlooked at the time of the first injury or which may develop in the subsequent process were not found to seriously affect the functional outcomes of tibial plateau fractures. It can be considered that even if a ligament injury is seen to be accompanying the fracture at the time of the first trauma, the effect is not great in terms of prognosis, and the treatment can remain conservative, especially for collateral ligament injuries.

## Introduction

Tibial plateau fractures are one of the most complex and common intra-articular fractures encountered in trauma surgery. These occur in young patients as a result of high-energy trauma and in the elderly with low-energy injuries, and constitute approximately 1% of all fractures [1]. As these are intra-articular fractures, complications may develop such as instability, infection, compartment syndrome, stiffness, and post-traumatic arthrosis [2]. These have a significant negative effect not only on the surgery but also on quality of life and functional independence. Deterioration of the functional integrity of the knee and the duration away from work cause a significant socioeconomic effect. Tibial plateau fractures are seen more often between the ages of 40 and 60 years and research has shown that the injury mostly (55-70%) affects the lateral plateau. Isolated injuries in the medial plateau are seen in 10-25% of cases, and bicondylar fractures involving both plateaux have been found in approximately 15% of reported series [3].

The main aim of surgical treatment is the anatomic restoration of condyle width and joint surface after fixation [4]. With the underlying effect of high-energy trauma, soft tissue injuries usually accompany tibial plateau fractures. Studies in the literature that have been conducted with postoperative magnetic resonance imaging (MRI) have determined a high rate of meniscus injuries, followed by anterior and posterior cruciate ligament injuries [5]. There have been shown to be significant accompanying soft tissue injuries in Schatzker type IV-VI injuries, which are high-energy, compared to low-energy type I-III injuries [6]. The incidence of secondary operations required to treat soft tissue injuries is not clear.

The aim of this study was to diagnose soft tissue injuries with MRI following surgically treated tibial plateau fractures and to evaluate how these injuries are reflected in the clinical outcomes.

## Materials and methods

Approval for this study was granted by the Ethics Committee, Süleyman Demirel University Medical Faculty (Approval No.: 352, dated November 17, 2020). The records were screened of 91 patients who underwent surgery for a tibial plateau fracture between January 2010 and November 2019. The study inclusion criteria were defined as age ≥18 years, no history of ipsilateral tibia fracture, no neurological or vascular problems in the affected extremity before the fracture, regular attendance at follow-up appointments, complete records of imaging after the trauma, no preoperative meniscus or ligament damage that required intervention, and a follow-up period of at least 12 months. Patients were excluded from the study if they had another injury on the ipsilateral extremity. The demographic data, medical comorbidities, mechanism of injury, and clinical follow-up data were retrieved from the medical records of the patients. 

Two-directional anteroposterior (AP) and lateral radiographs and computed tomography (CT) scans were taken of the knee preoperatively to determine fracture severity and to plan the surgery. The surgical stabilization decision was determined according to a step-off of ≥2mm and the amount of expansion in the joint surface. Surgical success was defined as achieving anatomic alignment and joint congruence. Ligamentous laxity and instability were evaluated intraoperatively. The fractures were evaluated according to the Schatzker classification [7]. At the final follow-up examination, two-directional radiographs and non-contrast MRIs were taken of the operated knee. The MARS (metal artifacts reduction sequence) protocol was applied in the MRI. In the general evaluation of the knee joint with MRI, the semi-quantitative Whole-Organ Magnetic Resonance Imaging Score (WORMS) was used as the scoring system [8]. Postoperatively, an angle-adjustable knee brace was applied to all patients and a standard rehabilitation protocol by physiotherapists.

The Knee Society Knee Score (KSKS) and Knee Society Function Score (KSFS) were used for clinical scoring [9]. In addition, the Knee Injury and Osteoarthritis Outcome Score (KOOS) was used for the subjective evaluation of symptoms associated with knee injuries and knee osteoarthritis. For the radiological evaluation, postoperative reduction was evaluated using the Rasmussen radiological scoring system on the postoperative radiographs of the patients [10].

Statistical analysis

Data obtained in the study were analyzed statistically using the statistical software IBM SPSS Statistics for Windows, Version 22 (IBM Corp., Armonk, NY). Descriptive statistics were stated as mean ±standard deviation (SD) values for continuous variables and as number (n) and percentage (%) for categorical variables. The Chi-square test was used in the analysis of categorical variables. In the analysis of continuous variables, the non-parametric Mann-Whitney U-test was applied to two independent groups of data not showing normal distribution, and for three or more independent groups, the Kruskal-Wallis test was used. Spearman correlation analysis was applied in the evaluation of relationships between continuous variables. In all the tests, a value of p<0.05 was accepted as statistically significant.

## Results

Evaluation was made of a total of 49 patients, comprising 39 (79.6%) males and 10 (20.4%) females with a mean age of 45.9±11.89 years. 

The trauma etiology was seen to be 63.2% traffic accident outside a vehicle, 12.2% traffic accident within a vehicle, 8.2% fall from height, 10.2% workplace accident, and 6.2% simple fall. An open fracture was determined in seven (14.2%) patients. All of the open fractures were Schatzker type VI fractures. According to the Gustilo-Anderson classification, one of these seven patients had type 2 fracture, three patients had type 3A, one patient had type 3B, and two patients had type 3C. Compartment syndrome did not develop in any patient. Two-stage surgery was performed on four patients.

When the fracture types of the patients were examined, 10.2% (n=5) were type 1, 32.7% (n=16) were type 2, 6.1% (n=3) were type 3, 8.2% (n=4) were type 4, 14.3% (n=7) were type 5, and 28.6% (n=14) were type 6. It was determined that the fractures were in the lateral plateau in 49% (n=24), the medial plateau in 6.1%(n=4), and were bicondylar in 42.9% (n=21).

In the examination of complications, malalignment of >10° was seen in 8.2% (n=4), non-union in 6.1% (n=3), and postoperative infection in 6.1% (n=3). No complications developed in 79.6% of the patients.

The types of fixation applied to the patients were 44.9% (n=22) lateral plate, 18.4% (n=9) medial and lateral double plate, 12.2% (n=6) medial plate, 8.2% (n=4) cannulated screws only, 10.2% (n=5) temporary fixation with an external fixator followed by plate-screw osteosynthesis, and 6.1% (n=3) hybrid external fixator.

**Figure 1:**
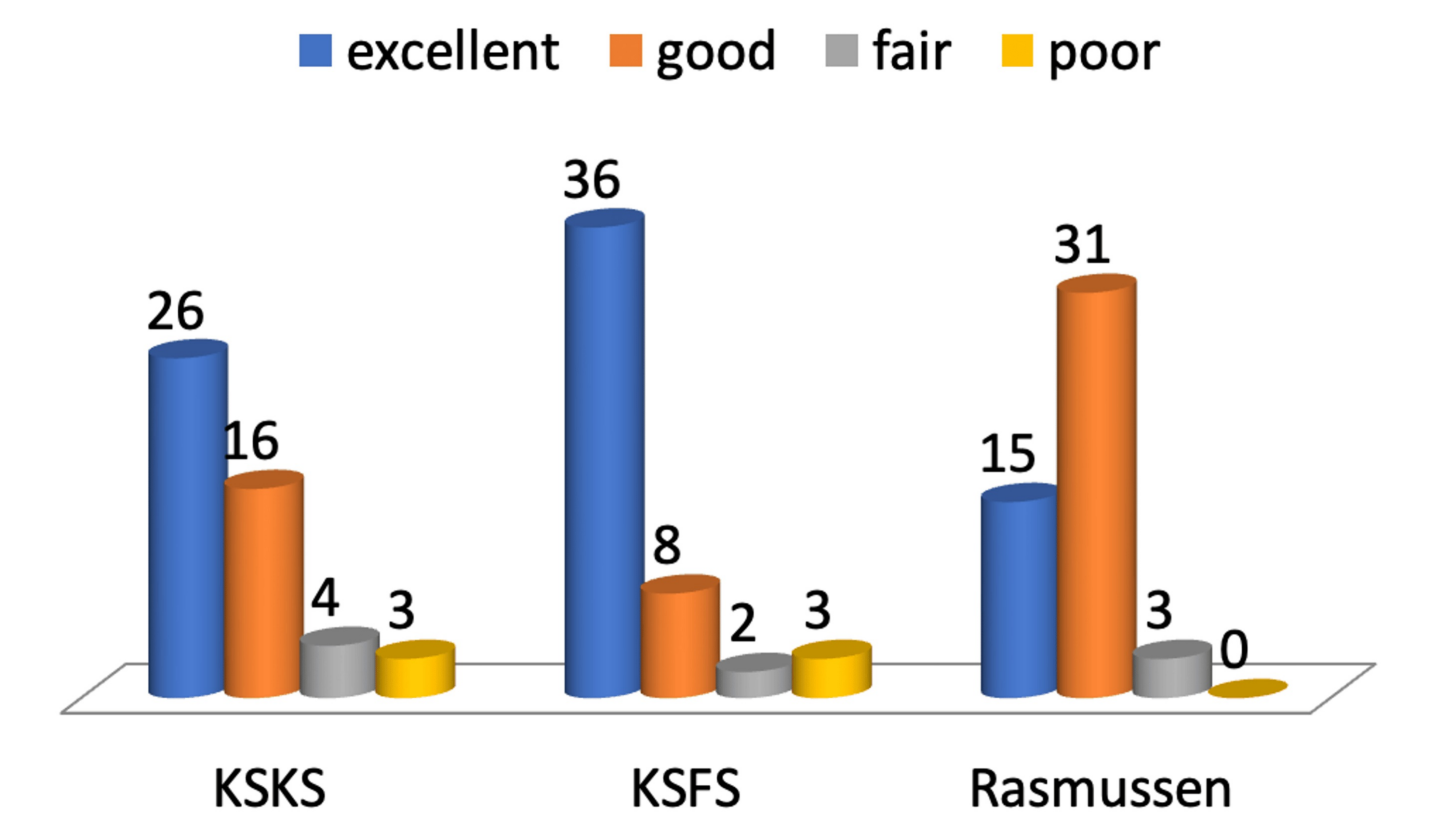
Distribution of the patients according to the KSKS, KSFS and Rasmussen scores. KSKS: Knee Society Knee Score, KSFS: Knee Society Function Score.

In the evaluation of the knee score with the KSKS, the mean score was determined as 86.7±15 (range, 45-100). The scores were evaluated as excellent in 26 patients, good in 16, fair in 4, and poor in 3. The results were satisfactory in 42 (85.7%) patients. In the evaluation of the knee functional score with the KSFS, the mean score was determined as 91.3±17.1 (range, 30-100). The scores were evaluated as excellent in 36 patients, good in eight, fair in two, and poor in three. The results were satisfactory in 44 (89.7%) patients (Figure 1). 

When examined according to the fracture types, statistically significant differences were determined in the WORMS points for cartilage (p<0.05), osteophytes (p<0.05), and bone wear (p<0.05).

A statistically significant difference was determined between the low-energy fracture (type 1-3) and high-energy fracture (type 4-6) groups in respect of the WORMS cartilage points (z=-2.106, p<0.05) and WORMS osteophyte points (z=-3.005, p<0.05) In the Schatzker type 1-3 low-energy fractures, the WORMS cartilage, osteophyte and subarticular cyst points were found to be statistically significantly lower (Table 1).

**Table 1:** Scores obtained from the WORMS subtypes as low-energy fracture type (1-2-3) or high-energy fracture type (4-5-6), and the Mann Whitney U-test results. n: number of patients, z: standard score, p: p value, *: significant at the 0.05 level according to Mann-Whitney U analysis. WORMS: Whole-Organ Magnetic Resonance Imaging Score.

WORMS	Schatzker type	n	Z	p
Cartilage	1-2-3	24	-2.106	0.035*
Cartilage	4-5-6	25		
Osteophytes	1-2-3	24	-3.005	0.003*
Osteophytes	4-5-6	25		
Bone wear	1-2-3	24	-1.649	0.099
Bone wear	4-5-6	25		
Meniscus	1-2-3	24	-0.501	0.616
Meniscus	4-5-6	25		
Ligament	1-2-3	24	-0.812	0.417
Ligament	4-5-6	25		
Synovitis	1-2-3	24	-0.380	0.704
Synovitis	4-5-6	25		
Bone marrow edema	1-2-3	24	-1.667	0.095
Bone marrow edema	4-5-6	25		
Subarticular cyst	1-2-3	24	-2.665	0.008*
Subarticular cyst	4-5-6	25		

In 41 of the 49 patients, a lateral and/or medial meniscus lesion was determined. No pathology in the meniscus was determined in eight patients. The lateral meniscus was determined to have a tear in 21 patients, degeneration without any tear in 17, and was evaluated as normal in 11. The medial meniscus was determined to have a tear in 11 patients, degeneration without any tear in 21, and was evaluated as normal in 17. In 16 patients, signal increase in the anterior cruciate ligament (ACL) and sprain findings were seen, ACL full thickness rupture in three, and posterior cruciate ligament (PCL) sprain findings in one. An increase in medial collateral ligament (MCL) thickness was seen in 15 patients, lateral collateral ligament (LCL) signal increase in one, and LCL elongation in one. No full thickness or partial rupture was observed in any collateral ligament.

## Discussion

The largest and kinematically most complex joint in the body is the knee joint, and the main weight-bearing surface within this joint is the tibial plateau. Tibial plateau fractures occur following high-energy trauma and generally affect young individuals [1]. Therefore, soft tissue problems in these fractures, the development of osteoarthritis after the fracture, and secondary pathologies seen in the meniscus, cartilage, and ligaments, and the management of these is important.

According to the MR images taken of the patients at the final follow-up examination, the mean total WORMS score was 34.2±28.3, with lowest score of 4 and highest of 129. Pathological changes were seen on the MRI of all the patients. No statistically significant correlation was determined between the Rasmussen radiological evaluation results and the Kellgren-Lawrence osteoarthritis grade. The good or poor radiological results were always reflected in the same way as the clinical results. Just as advanced-grade osteoarthritis can develop despite a good radiological score, so osteoarthritis may not develop even when the radiological score is poor.

A statistically significant difference was determined in the WORMS (p<0.05) and KSKS (p<0.05) scores according to the Kellgren-Lawrence osteoarthritis grade. As the Kellgren-Lawrence grade increased, so there was seen to be an increase in the WORMS points, and as the Kellgren-Lawrence grade decreased, the KSKS points increased. Patients who did not develop osteoarthritis during the follow-up period according to the Kellgren-Lawrence grades, were seen to have clinically better knee scores and fewer pathologies within the knee as evaluated with WORMS. A statistically significant difference was seen between the Kellgren-Lawrence osteoarthritis grades with respect to the WORMS cartilage, WORMS osteophyte, WORMS bone marrow edema, WORMS subarticular cyst, and WORMS bone wear points (p<0.05).

In the treatment of tibial plateau fractures, the question of which patients should be treated conservatively and which, surgically, remains a matter of debate, and no consensus has been reached on the subject of indications. Some authors have reported that independently of fracture type, satisfactory results have been obtained with conservative treatment, including full anatomic reduction. Apley et al. reported satisfactory results in patients who underwent conservative treatment of traction and early joint movement with a continuous passive motion (CPM) device [11]. Duwelius et al. applied brace treatment following closed reduction and then early mobilization according to traction treatment, and the outcomes were found to be satisfactory [12]. However, it must not be forgotten that although a good result was obtained in 83 patients in terms of walking capacity, the same success was not obtained in joint range of movement, and flexion limitation can be seen. It has also been stated that varus or valgus deformities, depending on the fracture type, can develop in the long term [13].

When the ligaments were examined in the current study, signal increase in the ACL and sprain findings were seen in 16 patients, full-thickness ACL rupture in three, and PCL sprain findings in one. In the collateral ligaments, there was seen to be increased MCL thickness in 15 (30%) patients, LCL signal increase in one, and LCL elongation in one. No full thickness or partial rupture was observed in any collateral ligament. Ligament abnormalities were identified on MRI in a total of 28 (57%) patients. Full-thickness ACL rupture was determined in three patients. Thickening of the MCL was seen in a third of the study patients, and in half of the patients with Schatzker type 2 fractures. It is not surprising that collateral ligament injuries can form associated with the forces causing plateau fractures. Lateral plateau fractures due to valgus forces can be associated with MCL injuries. In the past, collateral ligament injuries were thought to be more common because of instability in the first examination. This instability can also occur due to collapse of the lateral plateau joint surface causing loss of bone support. Loss of bone support on one side of the joint actually protects the collateral ligament on the contralateral side.

An experimental study showed that an intact MCL, functioning as a hinge point for the lateral femoral condyle, is required to form a lateral plateau fracture [14]. The results of the current study suggest that MCL thickening could be associated with the fracture mechanism. In addition, the bones remaining in malalignment after surgical treatment can increase stress loading on ligaments and can explain the high rates of ACL and collateral ligament abnormalities seen. The treatment of ligament injuries accompanying tibial plateau fracture is a matter of debate. Gardner et al. showed that with a postoperative protocol of a hinged knee brace and non-weight-bearing on the extremity, MCL ruptures can recover sufficiently without acute surgical repair [5].

LCL rupture has been accepted as a relatively stabilized injury and despite limited outcome data, acute repair at the same time as the fracture has been advocated [15]. As reconstruction of ACL or PCL rupture during fracture fixation is extremely difficult, it is recommended that cruciate ligament tears are re-evaluated after fracture healing. No patient in the current study had an MCL or LCL rupture that required repair. In three patients with ACL rupture, no laxity in the knee was observed and there was no need for ACL reconstruction. Therefore, it was thought that even if a ligament injury is seen to be accompanying the fracture at the time of the first trauma, the effect is not great in terms of prognosis, and the treatment can remain conservative, especially for collateral ligament injuries.

One of the limitations of the study is the small number of patients. Another issue is the lack of a longer-term and comprehensive study. Despite everything, we think that our study will make an important contribution to the current literature. Studies on this subject have been very rare.

## Conclusions

The results of this study showed that meniscus injuries which are overlooked at the time of the first injury or which may develop in the subsequent process were not found to seriously affect the functional outcomes of tibial plateau fracture. Therefore, it was thought that even if a ligament injury is seen to be accompanying the fracture at the time of the first trauma, the effect is not great in terms of prognosis, and the treatment can remain conservative, especially for collateral ligament injuries.
